# Characteristics and racial variations of polypoidal choroidal vasculopathy in tertiary centers in the United States and United Kingdom

**DOI:** 10.1186/s40942-017-0060-4

**Published:** 2017-04-17

**Authors:** Tarek Alasil, Nelida Munoz, Pearse A. Keane, Adnan Tufail, Patrick A. Coady, Eduardo Novais, Talisa E. de Carlo, Caroline R. Baumal, Nadia K. Waheed, Jay S. Duker, Ron A. Adelman

**Affiliations:** 1 Department of Ophthalmology and Visual Sciences, School of Medicine, Yale University, New Haven, CT USA; 2 Moorfields Eye Hospital NHS Foundation Trust, UCL Institute of Ophthalmology, London, UK; 3 New England Eye Center, Tufts Medical Center, Boston, MA USA; 4 School of Medicine, Federal University of São Paulo, São Paulo, Brazil

**Keywords:** Polypoidal choroidal vasculopathy (PCV), Indocyanine green angiography (ICGA), Fluorescein angiography (FA), Optical coherence tomography (OCT)

## Abstract

**Purpose:**

To evaluate the characteristics and racial variations amongst patients with polypoidal choroidal vasculopathy (PCV) in the United States and the United Kingdom.

**Methods:**

Fundus photos and indocyanine green angiography images were evaluated in a multicenter retrospective study to establish the diagnosis of PCV. Visual acuity (VA) was recorded in ETDRS letter count.

**Results:**

Eighty eyes of 71 PCV patients (average age of 69.4 ± 10.4 years) were included in the analysis. Of the total 71 subjects, 46 (65%) were women, 33 (46.5%) were Blacks, 16 (22.5%) were Whites, 19 (26.8%) were Asians and 3 (4.2%) belonged to other races. The Black subgroup had vision gain of 3.5 letters. The White and Asian subgroups had vision loss of 13.1 and 3.5 letters, respectively. There was female predominance in Blacks (67%), Whites (69%), and Asians (58%). PCV was found to be a bilateral disease in 14 patients (20%). There was significant decrease of 7 letters with every decade increase in age (p = 0.005). Final VA was worse in males when compared to females (p = 0.042), and worse in Whites when compared to Blacks (p = 0.005). For every 10 letters worse in initial VA upon diagnosis with PCV, the final VA was worse by 6 letters (p < 0.001). The location of the polypoidal lesion within the macula was associated with significant decrease of 14 letters in BCVA (p = 0.02). The length of follow up was significantly associated with worse visual outcome (p = 0.012). Final VA had no significant correlation with the lens status, or the different treatment modalities.

**Conclusions:**

Based on our cohort from tertiary centers in the United States and United Kingdom, PCV is a bilateral disease in one-fifth of patients. It features a variable female predominance based on ethnicity. Increased age, worse vision upon initial presentation, longer follow up and macular location of the polyp were associated with worse visual outcome.

**Electronic supplementary material:**

The online version of this article (doi:10.1186/s40942-017-0060-4) contains supplementary material, which is available to authorized users.

## Background

Polypoidal choroidal vasculopathy (PCV) is a distinct clinical entity that is associated with abnormal inner choroidal vascular network of vessels ending in an aneurysmal projection, visible clinically in some cases as orange reddish spheroid polyp-like structures [1]. PCV is characterized by multiple, recurrent, serosanguinous detachments of the retinal pigment epithelium and neurosensory retina secondary to leakage and bleeding from the polypoidal lesions [1, 2].

Most of the large PCV studies come from Japan, South Korea and Singapore, where the disease has been reported to be more prevalent. Japanese studies have shown that one-fourth to one half of the elderly patients with neovascular age-related macular degeneration (AMD) in Japan were diagnosed with PCV [3, 4]. However, individuals of other ethnicities may develop PCV as well. Lafaut et al. [5] reported ICGA-confirmed PCV in 4% of white patients with occult choroidal neovascularization. Recently, a Swiss study revealed PCV in 16.8% of Caucasian patients with neovascular AMD [6].

Indocyanine green angiography (ICGA) remains the gold standard in establishing the diagnosis of PCV, because it can demonstrate the polypoidal lesions and the branching vascular network underneath the RPE, which can be missed using standard fluorescein angiography [7]. However, it is important to realize that not all focal subretinal nodular hyperfluorescence on ICGA is presumed to be due to polyps. Therefore, the EVEREST study report 2 described the imaging and grading protocols to standardize the diagnostic criteria for future randomized controlled trials on PCV [8]. Multiple reports have described the optical coherence tomography (OCT) features of PCV and correlated them with ICGA findings [9–17].

The natural course of PCV usually follows a relapsing-remitting course, where the visual outcome can vary. Uyama et al. [18] followed 14 eyes of 12 patients with PCV for 2 years without any treatment. A favorable outcome was noted in 50% of patients, whereas the remaining half suffered from persistence of the disease and repeated serosanguinous leakage, resulting in unfavorable visual outcome. On the other hand, the treatment guidelines for PCV were established by the Everest Study Group (a prospective multicenter randomized controlled clinical trial, which included 61 Asian patients) which recommended treatment of juxtafoveal and subfoveal PCV with ICGA-guided photodynamic therapy (PDT) or PDT plus 3 ranibizumab intravitreal injections 1 month apart [19, 20]. Recent studies have observed and reported resistance to ranibizumab in some patients with PCV [21, 22]. There have been recent studies from Japan reporting the use of aflibercept in PCV patients, who are naïve to treatment or not responding to ranibizumab [23–25]. The long term behavior of visual acuity (VA) in PCV patients of different ethnicities remains controversial and further studies are warranted. Our study describes the characteristics and visual outcomes in PCV patients of different ethnicities from three tertiary centers in the United States and the United Kingdom.

## Methods

### Data collection

We retrospectively reviewed the records of patients with PCV at Yale Eye Center, Moorfields Eye Hospital and New England Eye Center between September 2005 and December 2015. Study protocols were approved by the Institutional Review Board at Yale University and Tufts University and were in accordance with the Health Insurance Portability and Accountability Act. The research adhered to the tenets of the Declaration of Helsinki for research involving human subjects.

After comprehensive chart review, 155 eyes with PCV were identified in a retrospective multicenter study. The VA score from the United Kingdom (Moorfields) was recorded using the Early Treatment Diabetic Retinopathy Study (ETDRS) letter count. The VA score from the United States centers (Tufts University and Yale University) was recorded using Snellen charts, then converted to ETDRS letter count using a conversion chart. The VA for all patients was documented at baseline and last follow up visit. Age at the time of initial diagnosis, ethnicity, and lens status (phakic versus pseudophakic) were documented. Dilated fundus examination findings and color fundus photography images were reviewed. Patients with concurrent retinal pathologies were excluded from the analysis.

**Fig. 1:**
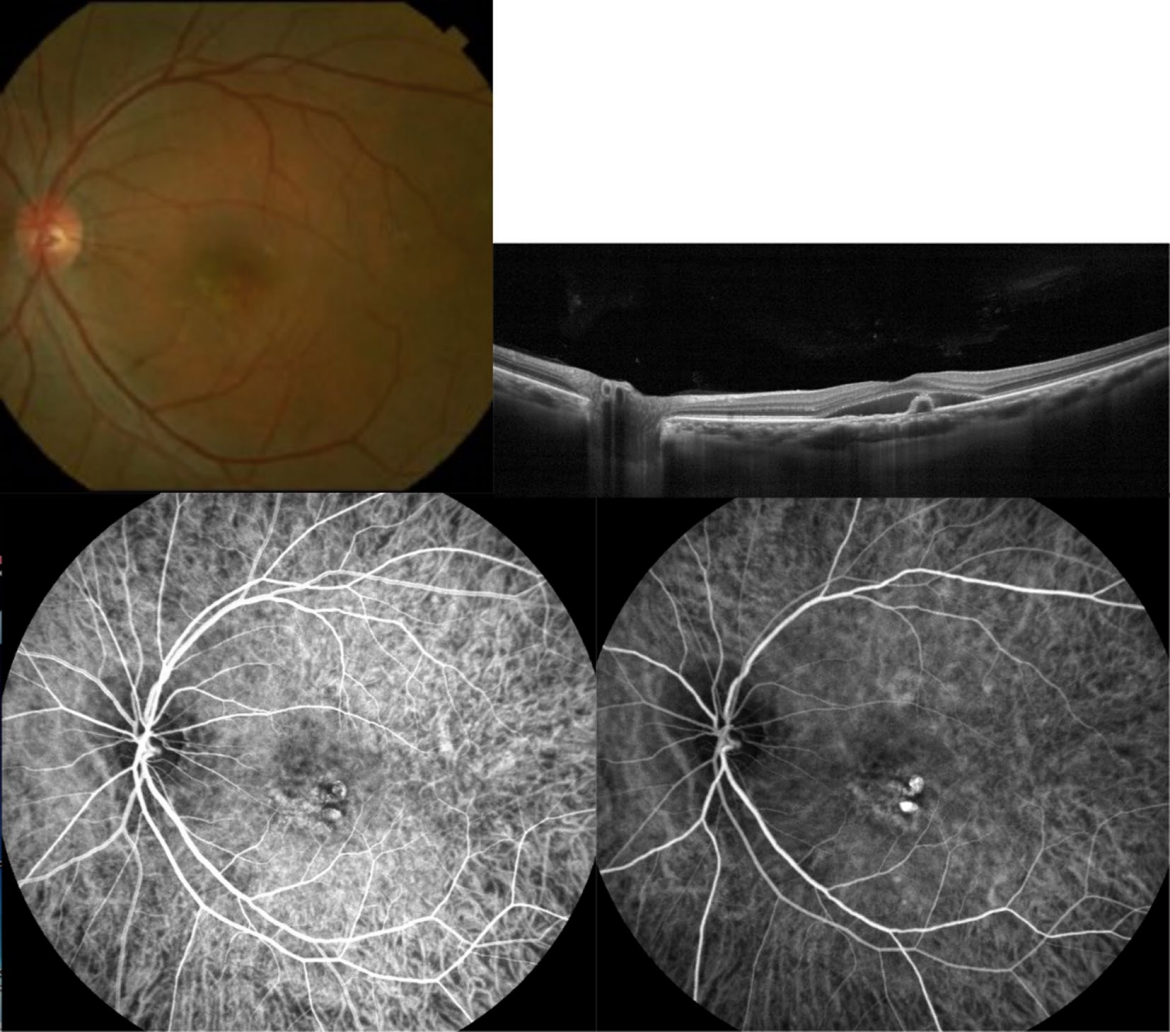
The EVEREST study diagnostic criteria for polypoidal choroidal vasculopathy. *Upper row Color* fundus photograph of the left eye of a 71 year old Asian man shows nodular appearance of the central macular polyp. Cross-sectional OCT shows central pigment epithelial detachment (where the polyp is located) with surrounding subretinal fluid. *Bottom row* Early and late frames from dynamic indocyanine green angiography (ICGA) show progressive filling of the central polyps and hypofluorescent halo around the polyp. Additional file 1: Video 1 demonstrates the dynamic ICGA

**Fig. 2:**
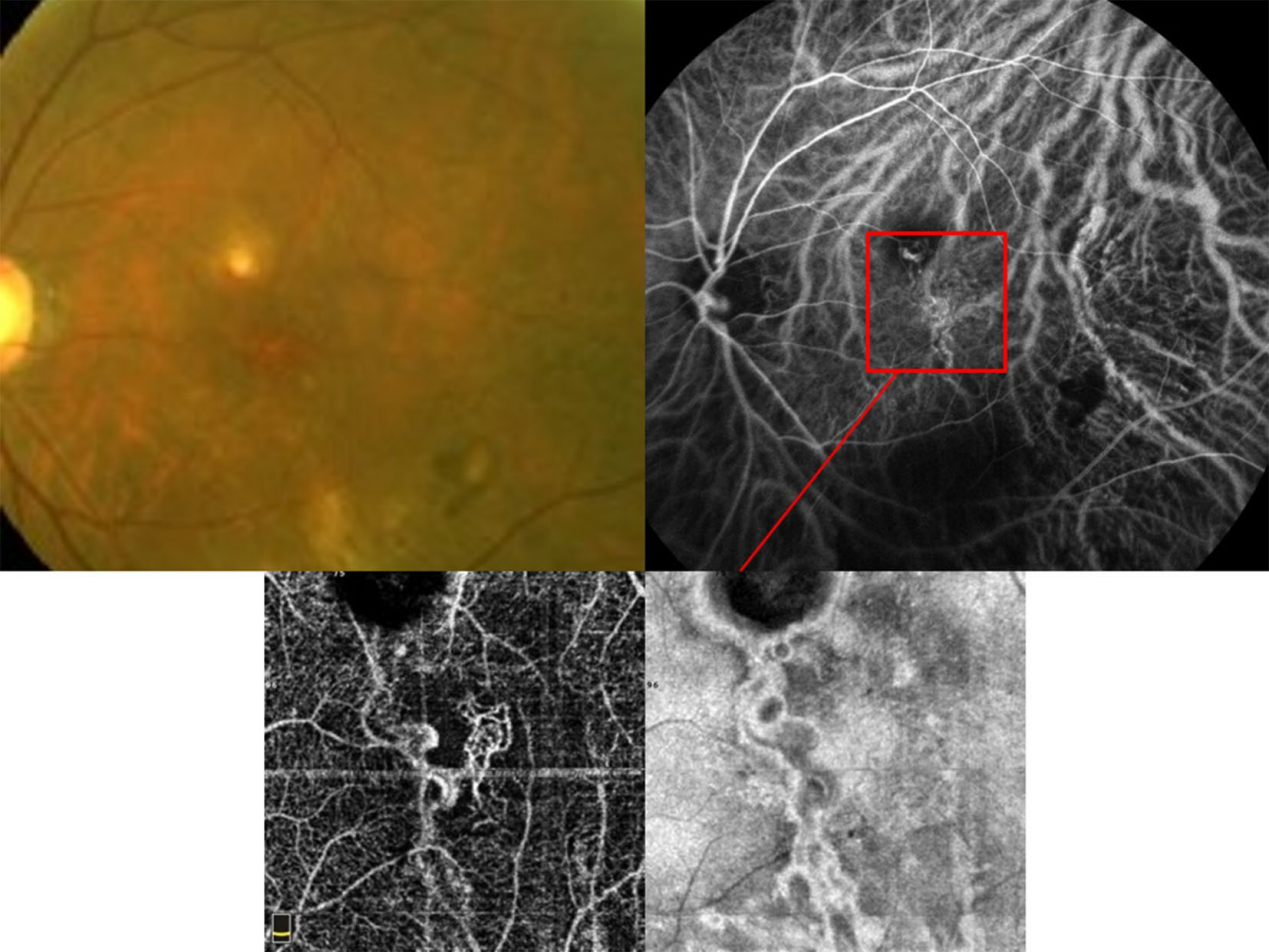
The EVEREST study diagnostic criteria for polypoidal choroidal vasculopathy. Color Fundus photograph of the left eye of a 61 year old Asian female shows orange subretinal nodular appearance of a central macular polyp (superior to the fovea). Early indocyanine green angiography shows partial filling of the polypoidal lesion, hypofluorescent halo around the polyp, and a branching vascular network (inferior to the polyp). The branching vascular network (red aquare) is further demonstrated using AngioVue optical coherence tomography angiography system (Optovue, Inc, Fremont, CA); en face images of the choriocapillaris slab

PCV diagnosis was confirmed based on the EVEREST definition [8, 26, 27], including the presence of early focal subretinal hyperfluorescence on ICGA and at least one of the following six diagnostic criteria: (1) nodular appearance of polyp(s) on stereoscopic examination, (2) hypofluorescent halo around nodule(s), (3) presence of a branching vascular network, (4) Pulsatile filling of the polyps on dynamic ICGA, (5) orange subretinal nodules on color fundus photography, or (6) massive submacular hemorrhage (≥4 disc areas in size). Figures 1, 2 3 and Additional file 1: Video 1 further demonstrate some of the aforementioned diagnostic features.

**Fig. 3:**
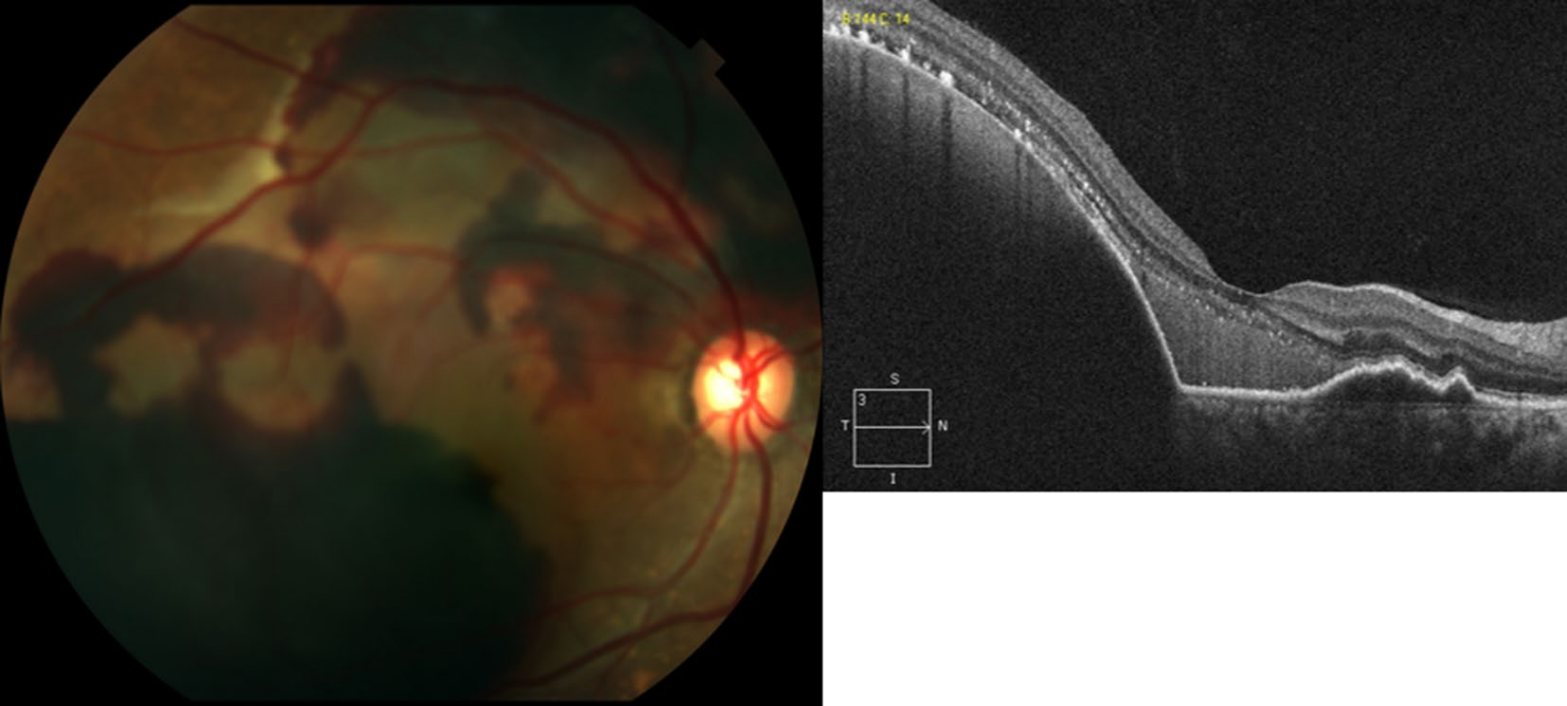
The EVEREST study diagnostic criteria for polypoidal choroidal vasculopathy. Color fundus photograph of the right eye of a 56 year old Black man shows massive submacular hemorrhage (≥4 disc areas in size). Corresponding cross-sectional optical coherence tomography shows large pigment epithelial detachment along the temporal macula consistent with sub-macular hemorrhage

**Fig. 4:**
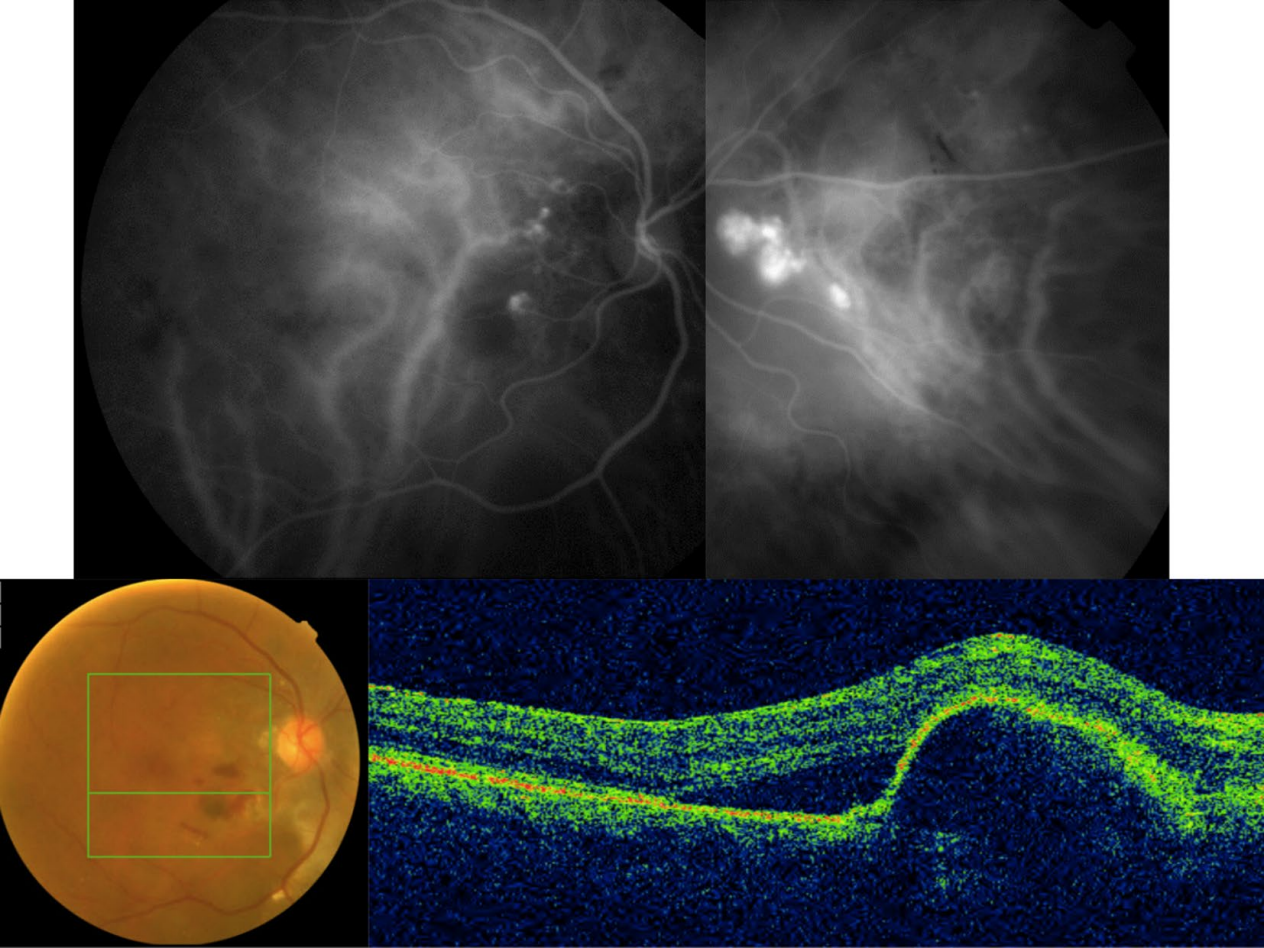
Multimodal imaging of the right eye of a 76-year-old Black female with polypoidal choroidal vasculopathy (PCV) who was followed for 56 months. Best corrected visual acuity (BCVA) upon initial diagnosis with PCV was 20/125 (61 letters), and worsened to counting fingers at last follow up visit. Treatment included 6 intravitreal injections with ranibizumab. Indocyanine green angiography image is showing late choroidal staining consistent with polypoidal lesions within the macula and nasal to the optic nerve. *Upper row*: Indocyanine green angiography imaging demonstrated macular and extramacular polypoidal lesions. *Lower row*: Color fundus photograph shows orange subretinal nodule, intraretinal and subretinal hemorrhage. Optical coherence tomography cross sectional B-scan through the macular polypoidal lesions (upon presentation) is demonstrating a large serous pigment epithelial detachment (PED) within the macula with associated subretinal fluid temporally and small temporal notch

**Fig. 5:**
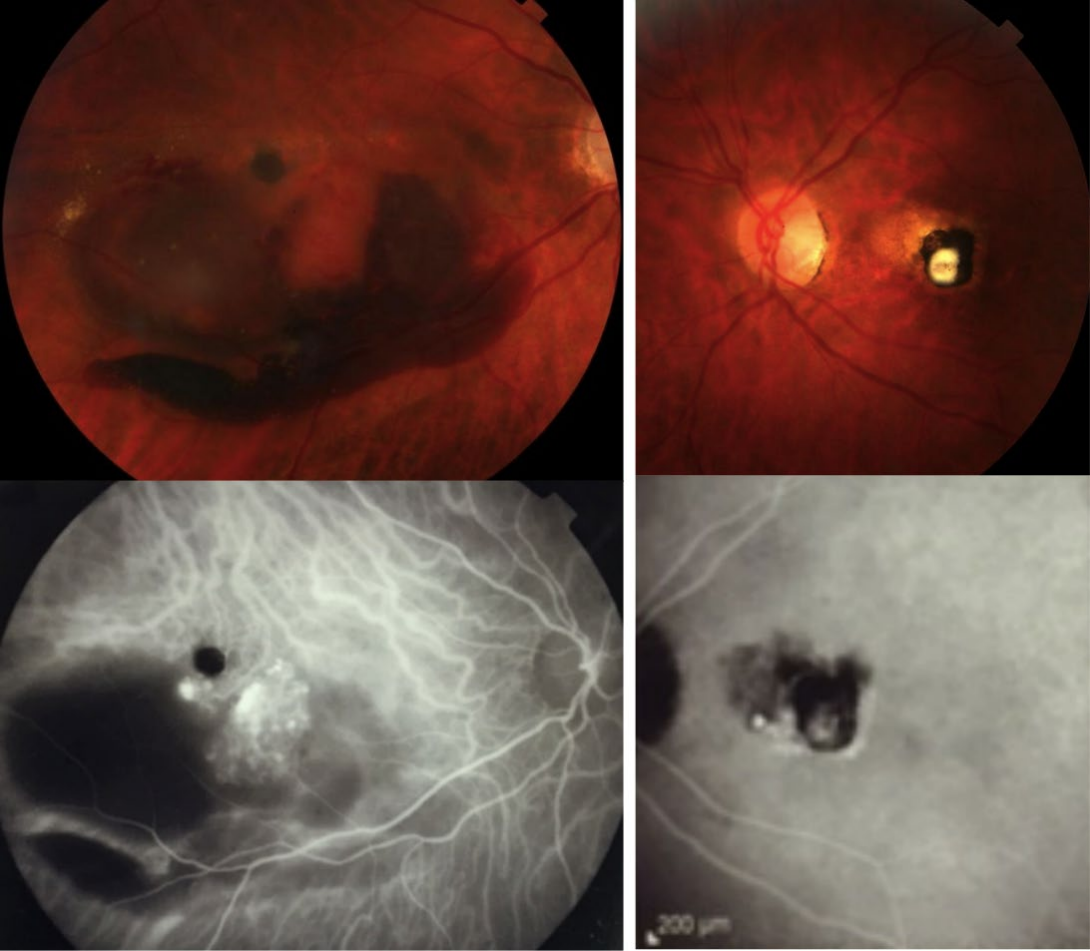
Fundus photographs and indocyanine green angiography (ICGA) imaging of a 50-year-old white woman with metabolic syndrome and bilateral polypoidal choroidal vasculopathy (PCV) who was followed for 104 months. Fundus photos show subretinal hemorrhage along the inferotemporal macula and inferotemporal arcade OD, and central macula scar with pigmentation OS. ICGA shows polypoidal lesions along the temporal macula adjacent to the area of submacular hemorrhage OD and a polyp along the nasal edge of the central macula scar OS. Best corrected visual acuity (BCVA) upon initial diagnosis with PCV was 20/100 (65 letters) OD and counting fingers OS. Vision improved to 20/50 (80 letters) at last follow up visit OD after treatment with focal laser plus 12, 5 and 9 intravitreal injections with bevacizumab, ranibizumab and aflibercept, respectively. Left eye received no treatment and vision remained at counting fingers

**Fig. 6:**
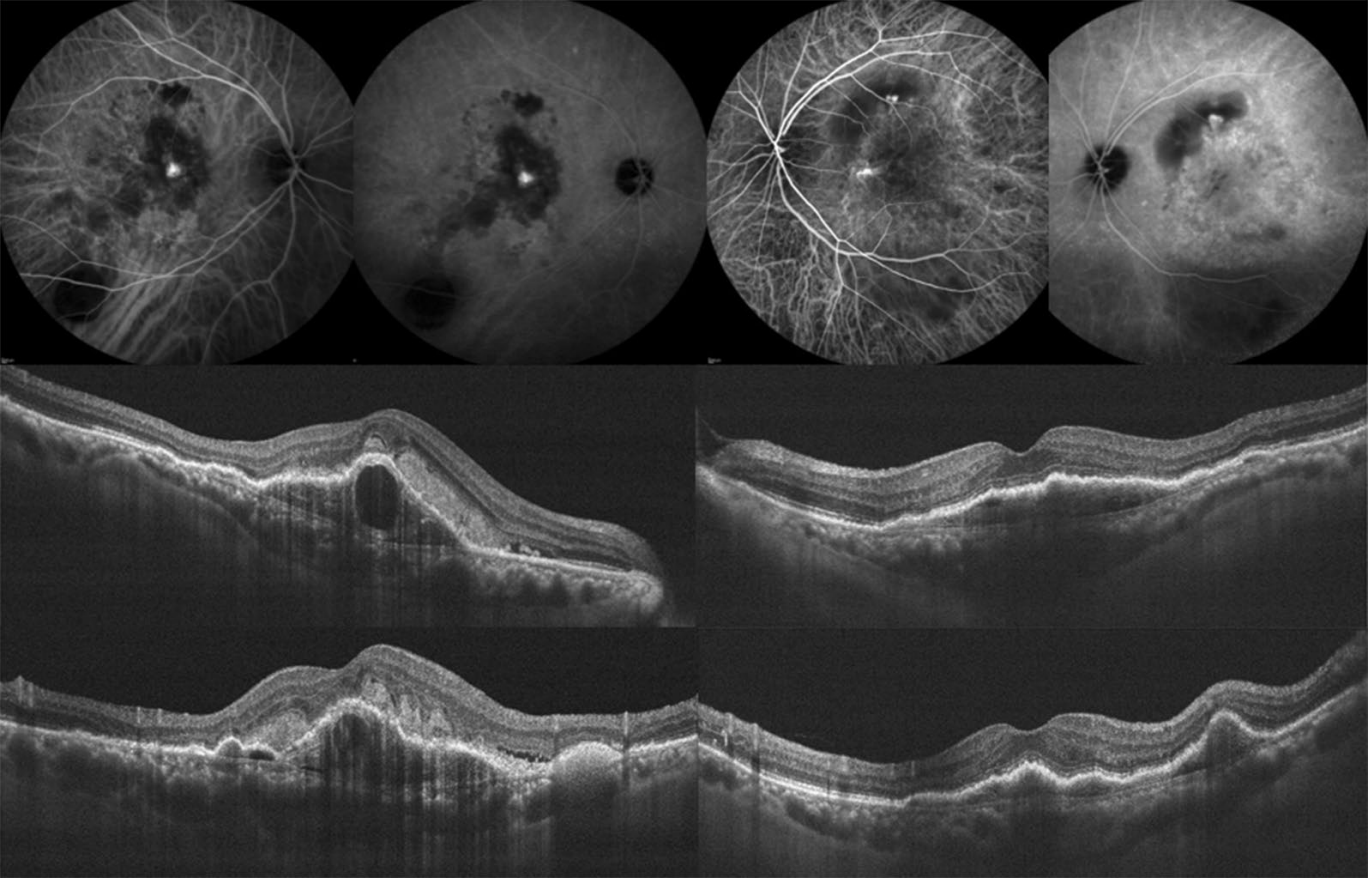
Indocyanine green angiography (ICGA) and optical coherence tomography (OCT) imaging of a 71 year old Asian man with bilateral polypoidal choroidal vasculopathy (PCV) who was followed for 35 months. Early and late ICGA images show central macular polyp OD and superior macula polypoidal lesion with central macular choroidal vascular dilation OS. Cross-sectional OCT scan images show pigment epithelial detachments, double layer sign (branching vascular network) and thick choroid in both eyes. Best corrected visual acuity (BCVA) upon initial diagnosis with PCV was 65 letters OD and 57 letters OS. Vision worsened to 12 letters at last follow up visit OD after treatment with 4 and 9 intravitreal injections with bevacizumab and aflibercept, respectively. Left eye received 4 and 10 intravitreal injections with bevacizumab and aflibercept respectively and vision improved to 66 letters

ICGA confirmed the presence of the polyps (Figs. 1, 4, 5, 6). The location of the polyps was recorded as either macular or extra-macular (based on the anatomical macula with a diameter of 5.5 mm centered at the fovea). In eyes where both macular and extramacular polypoidal lesions were observed simultaneously, eyes were labeled as having macular polypoidal lesions (Fig. 4). OCT images were evaluated for the following features: pigment epithelial detachment (PED), subretinal fluid (SRF), branching vascular network (BVN) and intraretinal fluid (IRF). Figures 1, 3, 4, 6 show some of the abovementioned OCT features.

History of previous treatments with focal laser, PDT, intravitreal injections with anti-VEGF agents (bevacizumab, ranibizumab and aflibercept) were quantified and recorded in a chronological order.

Morphological features of PCV were identified by 2 independent authors (TA and NM) using a standard protocol to analyze color photographs, ICGA and OCT images. Disagreement between the two observers was resolved by open adjudication.

### Statistical methods

The variables data are presented as mean ± standard deviation. Multivariate analysis model was performed to determine the effects of age, gender, ethnicity, BCVA upon initial presentation, length of follow up, and different treatment modalities on the final BCVA in patients with PCV. p values of <0.05 were considered statistically significant.

## Results

### Patient characteristics

The 71 subjects included in the study had an average age of 69.4 ± 10.4 years (range 50–90 years old). Forty-seven subjects were from the United Kingdom (where VA was reported in ETDRS letter count), and 24 subjects were from the United States (where VA was reported in Snellen charts and converted to ETDRS letter count). The mean follow-up for the patients was 31.8 ± 27.9 months (range 2–120 months). Of the total 71 subjects, 46 (65%) were women, 33 (46.5%) were Blacks, 16 (22.5%) were Whites, 19 (26.8%) were Asians and 3 (4.2%) belonged to other races (Table 1). Out of the total 80 eyes included in the analysis, there were 38 right eyes and 42 left eyes.

**Table 1:** Demographics of subjects with polypoidal choroidal vasculopathy included in the analysis

Number of subjects	80 eyes (71 patients)	Mean age (years)	69.4 ± 10.4
Follow up (months)	31.8 ± 27.9		
Mean BCVA	Initial	Last follow up	
	65.1 ± 19.1	63.0 ± 27.6	
Mean change in BCVA	−2.1 ± 24.2 (ETDRS Letters)		
Gender	25 males (35%)	46 females (65%)	
Lens status	66 phakic eyes (82.5%)	14 pseudophakic eyes (17.5%)	

Ethnicity	Asian	Black	White	Other
Number of subjects	19 (26.8%)	33 (46.5%)	16 (22.5%)	3 (4.2%)
Gender (female/total)	11/19 (58%)	22/33 (67%)	11/16 (69%)	2/3 (67%)
Mean change in BCVA	−2.3 ± 29.0	3.5 ± 18.2	−13.1 ± 26.9	2.3 ± 10
Mean age (years)	67.8 ± 9.2	71.1 ± 9.8	67.8 ± 13.3	67.7 ± 6.8

BCVA indicates Best corrected visual acuity (ETDRS letter count)

PCV indicates polypoidal choroidal vasculopathy

### Location of the polypoidal lesions

Out of the total 80 eyes, 62 eyes had macular polypoidal lesions and 18 eyes had extra-macular lesions. Macular polypoidal lesions were observed in 25 out of 36 black eyes (69.4%), 18 out of 23 Asian eyes (78.3%), 16 out of 19 white eyes (84.2%) and in all 3 eyes of undocumented ethnicity. Those findings are summarized in Table 2.

**Table 2:** Location and laterality of polypoidal lesions on indocyanine green angiography of eyes with polypoidal choroidal vasculopathy included in the analysis

Ethnicity	Number of eyes	Location of polyps		Number of subjects	Bilateral disease
		Macular	Extra-macular		
Asians	23	18 (78.3%)	5 (21.7%)	19	4 (21%)
Blacks	36	25 (69.4%)	11 (30.6%)	33	7 (21%)
Whites	19	16 (84.2%)	3 (15.8%)	16	3 (19%)
Other	3	3 (100%)	0	3	0
Total	80	62 (77.5%)	18 (22.5%)	71	14 (20%)

Location of polypoidal lesions is based on indocyanine green angiography findings

### Optical coherence tomography imaging

Seventy-five eyes out of the total 80 eyes had OCT images that were available for analysis. Sixty-six eyes had either time-domain or spectral-domain OCT imaging, 7 eyes had prototype swept source OCT imaging and 2 eyes had AngioVue OCT angiography (Optovue, Inc, Fremont, CA). Out of the 75 eyes that had OCT imaging, 71 eyes had PED, 29 eyes had BVN, 65 eyes had SRF, and 18 eyes had IRF.

### Bilateral polypoidal choroidal vasculopathy

Based on ICGA findings in our study, and applying the abovementioned EVEREST study definitions, PCV was found to be a bilateral disease in 14/71 patients (20%). Out of the remaining 57 subjects with presumed unilateral PCV, one Asian patient had her fellow eye enucleated after a non-traumatic massive retinal hemorrhage, and another Asian patient had no light perception in her fellow eye (phthisis bulbi after untreated chronic hemorrhagic retinal detachment). Bilateral PCV was noted in 7/33 Black subjects (21%), 3/16 White subjects (19%) and 4/19 Asian subjects (21%). Table 2 summarizes these findings.

### Treatment

Five out of 80 eyes received no treatment at all (two eyes of two black patients gained 7 and 9 ETDRS letters respectively, 2 eyes of 2 white patients had no change in VA, and one eye of undocumented ethnicity lost 9 ETDRS letters at last follow up). Out of the remaining 75 eyes, 68 eyes received one or more intravitreal injections with anti-vascular endothelial growth factor agents (bevacizumab, ranibizumab, and or aflibercept). Twelve out of 75 eyes received one or more sessions of photodynamic therapy. Twenty-two out of 75 eyes received one or more sessions of focal laser treatment. There was one eye of a black patient which had received PDT, and intravitreal injections with bevacizumab and triamcinolone acetate. Pars plana vitrectomy was utilized after bevacizumab injections in one eye of a black patient, in order to clear massive subretinal hemorrhage and vitreous hemorrhage. Table 3 summarizes these findings.

**Table 3:** Treatment

	Number of eyes		
Total	80		
Observation (no treatment)	5		
Treatment	75		
Treatment modalities	PDT	12	
	Focal laser	22	
	Intravitreal injections with anti-VEGF agents	68	
	Total number of anti-VEGF injections		
	Bevacizumab	Ranibizumab	Aflibercept
	109 injections	369 injections	198 injections
	Intravitreal injection with triamcinolone	1	
	Pars plana vitrectomy	1	

*PDT* photodynamic therapy, *VEGF* vascular endothelial growth factor

### Multivariate analysis

There was a significant decrease in final VA of seven ETDRS letters with every decade increase in age starting at 50 years (p = 0.005). Final VA was worse in males when compared to females (p = 0.042). Final VA was significantly worse (by 18 letters) in Whites when compared to Blacks (p = 0.005). For every 10 letters worse in initial BCVA upon diagnosis with PCV, the final VA was worse by 6 letters (p < 0.001). The location of the polypoidal lesion within the anatomic macula was associated with significant decrease of 14 letters in BCVA (p = 0.02). The length of follow up was significantly associated with worse final visual outcome (p = 0.012). Final VA had no statistically significant correlation with the lens status (phakic versus pseudophakic), nor the different treatment modalities which the patients had received. The *R*-squared for our multiple regression analysis was 0.55 (55%), and the adjusted *R*-squared was 0.45 (45%). The multivariate analysis model results are further demonstrated in Table 4.

**Table 4:** Multiple linear regression of demographic and clinical variables: effect on best corrected visual acuity at last follow up in patients with polypoidal choroidal vasculopathy

Variables	Coefficients	Standard error	t Stat	p value	Lower 95%	Upper 95%
Age	−0.71	0.25	−2.89	0.005*	−1.21	−0.22
Gender^a^	−10.75	5.19	−2.07	0.042*	−21.12	−0.39
Ethnicity^b^						
Asian	−9.424398	6.08	−1.55	0.16	−21.56	2.71
White	−18.07	6.18	−2.92	0.005*	−30.41	−5.73
Other	0.58	13.06	0.045	0.97	−25.51	26.67
Initial BCVA	0.58	0.14	4.27	<0.001*	0.31	0.85
Follow up	−0.25	0.095	−2.6	0.012*	−0.44	−0.06
Polyp location	−14.41	6.00	−2.4	0.02*	−26.39	−2.42
Lens status	−0.96	6.94	−0.14	0.89	−14.83	12.91
Treatment						
PDT	−2.15	5.66	−0.38	0.71	−13.45	9.15
Focal laser	0.62	3.12	0.2	0.84	−5.61	6.84
Bevacizumab	1.94	0.96	2.03	0.05	0.034	3.85
Ranibizumab	0.65	0.4	1.64	0.11	−0.14	1.44
Aflibercept	0.60	0.79	0.76	0.45	−0.98	2.19

The dependent variable is best corrected visual acuity (BCVA) at last follow up visit documented as early treatment diabetic retinopathy study (ETDRS) letter count. The independent variables are age, gender, ethnicity, initial BCVA, follow up (months), polyp location, lens status, and different treatment modalities, including photodynamic therapy (PDT), focal laser, intravitreal injections with bevacizumab, ranibizumab, and aflibercept. R squared was 0.55 and adjusted R squared was 0.45

* Statistically significant *p* < 0.05

^a^Female was used as the reference group (dominant gender in our cohort)

^b^Black was used as the reference group (largest ethnic group in our cohort). All calculations were made based on a multivariate analysis model

## Discussion

The term PCV was first coined by Yannuzzi in 1982 (Yannuzzi LA. Idiopathic Polypoidal Choroidal Vasculopathy. Presented at the Macula Society Meeting in Miami, Florida). The features of PCV were later described in 1990 [1]. Kleiner and associates used the terminology “posterior uveal bleeding syndrome” to describe PCV [28]. Further understanding of this peculiar entity has matured over the past three decades. Yannuzzi and colleagues diagnosed PCV in 7.8% of 167 consecutive patients with presumed neovascularized AMD in the United States [29]. Recently, Kuroda et al. [30] reported PCV in 52.5% of 343 eyes with neovascular AMD in Japan. Our retrospective multicenter study investigated the characteristics and racial variations of PCV patients in three tertiary centers the United States and the United Kingdom, and evaluated the predictive factors that determine visual outcomes in those patients.

PCV involvement was reported to be mostly unilateral (90% of patients) in a large Japanese cohort of 100 patients with PCV [3]. Recently, Alasil et al. [9] demonstrated choroidal vascular abnormalities in the unaffected eyes of patients with PCV using en face prototype swept source OCT. In our cohort, PCV was found to be bilateral in about 20% of patients regardless of the ethnic subgroup. PCV is usually diagnosed in patients between the ages of 50 and 65 years old. The average age of onset reported in the literature for PCV patients was 60 years [2]. White patients tend to present with PCV at an older age [28]. The mean age in our cohort was 69 years old, which can be explained by the different ethnicities included in the sample, including 22.5% white individuals. Final VA was decreased by seven ETDRS letters for every decade increase in age starting at 50 years.

Male preponderance was reported in multiple Asian PCV studies, including 63% of 100 Japanese patients, [3] 78.5% of 79 Korean patients, [31] and 68.4% of 19 Chinese patients [32]. On the other hand, the initial posterior uveal bleeding syndrome report described predominance in women over men in a small sample of black patients [28]. de Mello et al. reported female preponderance (67% of 72 Brazilian patients) [33]. Hatz et al. [6] reported female preponderance (71% of 34 of Caucasian Swiss patients with PCV). Davis et al. [34] reported female preponderance of 52% in 27 white patients with PCV in the United States. In our cohort, PCV featured a variable female predominance based on ethnicity (58% in Asians, 67% in Blacks and 69% in Whites). Our multivariate analysis suggested that final VA could be worse in males when compared to females in PCV. However, the *p* value of 0.042, the small sample size and the 2:1 female to male ratio make those gender-related conclusions uncertain.

A panel of PCV experts analyzed the body of literature on PCV together with results of the Everest trial, and made recommendations for the management of PCV which were based on this analysis and expert opinions [20]. Delivering PDT to the polypoidal lesions will result in occlusion of the polypoidal lesions and focal choroidal ischemia at the treated areas [35, 36], which may result in further VEGF secretion from the surrounding intact branching vascular network and subsequent recurrence of serosanguinous exudation causing further visual deterioration. Hence, the benefits of combining PDT with intravitreal injections of anti-VEGF agents were strongly emphasized [20]. Focal laser can be utilized to target polypoidal lesions outside the central macula. However, it may cause progressive photoreceptor degeneration with fibrosis and atrophic changes in the area over the scar, resulting in no improvement or even worsening of VA. Our multivariate analysis showed no significant effects of different treatment modalities on BCVA, probably because our sample was mixed in regards to the treatments delivered to the patients, and our retrospective study was not designed to examine the effects of different treatment modalities on visual outcomes in patients with PCV. As expected, the location of the polypoidal lesion within the anatomic macula was associated with worse visual outcome.

Final VA was significantly worse (by 16 letters) in Whites when compared to Blacks, but was not statistically different when Asians were compared to Blacks. Blacks were used as the reference group, because they were the largest ethnic subgroup in our cohort. This might very well be a random finding despite the p value, given the relatively small sample size, the Black group had twice as much extra-macular lesions compared with the White group, which could explain the visual outcome differences. Nevertheless, PCV appears to have two forms: a subset of choroidal neovascularization from a variety of etiologies, most commonly related to neovascular age-related macular degeneration (AMD); or a different disease from AMD which is usually encountered in darkly pigmented individuals, and without other characteristic fundus findings of AMD. However, there appears to be some overlapping features between these two entities.

The baseline BCVA upon initial diagnosis with PCV and the length of follow up were both found to be important predictors of final VA. Traditionally, patients with active or poorly responding PCV lesions tend to follow longer than patients who respond well to treatment. The mean follow up for the patients in our cohort was 32 months, and the range varied between 2 and 120 months. The relapsing-remitting nature of PCV may explain the lengthy follow up in some of our patients.

Our study has a number of strengths; in particular, the ICGA-confirmed PCV patients included in this study met the EVEREST inclusion criteria, were diverse and probably representative of subjects with PCV seen in the United Kingdom and United States. The sample size was relatively small compared to the studies from Japan, South Korea and Singapore. However, different ethnicities were represented including Black, Asian and White individuals.

Our study also has a number of limitations related to its retrospective nature. There was variability in the follow up duration between patients. The centers participating in the study were tertiary referral centers. Our multivariate analysis *r*-squared result of 0.55 suggests that the model doesn’t explain well the observed data. The study was not specifically designed to evaluate the effects of ethnicity and different treatment modalities on the visual outcome in patients with PCV.

OCT angiography (OCTA) is a non-invasive imaging technology that can visualize the retinal and choroidal vasculature. This technology is increasingly being used to image CNV since it can visualize the neovascular complex both above and beneath the RPE [37, 38]. Figure 2 shows an example of OCTA imaging of polypoidal lesions and branching vascular network in PCV. Previous studies have shown that the branching vascular network can regularly be detected using OCTA [39, 40]. However, Kim et al. [39] showed that only 50% of the polyps could be identified using this imaging modality. It’s been hypothesized that a turbulent flow inside the polyp would not generate a decorrelation signal inside the threshold range of commercially available OCTA [40]. Therefore, we anticipate ICGA to remain the gold standard for making the diagnosis of PCV and guiding the PDT treatment when indicated. In the future, the speed and resolution improvements in OCT angiography might provide an additional diagnostic and monitoring tool in PCV.

## Conclusion

 Based on our cohort from tertiary centers in the United States and United Kingdom, PCV is a bilateral disease in 20% of patients. It features a variable female predominance based on ethnicity. The following co-variables are significantly associated with worse VA outcomes: increased age, worse vision upon initial presentation, longer follow up and macular location of the polyp.

## Additional file

**Additional file 1**. Video 1 is a supplement to Figure 1. Video 1 represents dynamic indocyanine green angiogrpahy imaging of the left eye. It demonstrates progressive filling of the polyps within the center of the macula.
